# Impact of different SARS-CoV-2 assays on laboratory turnaround time

**DOI:** 10.1099/jmm.0.001280

**Published:** 2021-05-06

**Authors:** Bastian Marquis, Onya Opota, Katia Jaton, Gilbert Greub

**Affiliations:** ^1^​ Institute of Microbiology, University Hospital Center and University of Lausanne, Lausanne, Switzerland

**Keywords:** COVID, diagnostic, NAAT, SARS-CoV-2, TAT, time to results

## Abstract

**Introduction:**

Clinical microbiology laboratories have had to cope with an increase in the volume of tests due to the emergence of the SARS-CoV-2 virus. Short turnaround times (TATs) are important for case tracing and to help clinicians in patient management. In such a context, high-throughput systems are essential to process the bulk of the tests. Rapid tests are also required to ensure shorter TATs for urgent situations. In our laboratory, SARS-CoV-2 assays were initially implemented on our custom platform using a previously published method. The commercial cobas 6800 (Roche diagnostics) assay and the GeneXpert Xpress (Cepheid) SARS-CoV-2 assay were implemented on 24 March and 8 April 2020, respectively, as soon as available.

**Hypothesis/Gap Statement:**

Despite the abundant literature on SARS-CoV-2 assays, the articles focus mainly on the diagnostic performances. This is to our knowledge the first article that specifically studies the TAT of different assays.

**Aim:**

We aimed to describe the impact of various SARS-CoV-2 assays on the TAT at the beginning of the outbreak.

**Methodology:**

In this study, we retrospectively analysed the TAT of all SARS-CoV-2 assays performed in our centre between 24 February and 9 June, 2020.

**Results:**

We retrieved 33 900 analyses, with a median TAT of 6.25 h. TATs were highest (6.9 h) when only our custom platform was used (24 February to 24 March, 2020). They were reduced to 6.1 h when the cobas system was introduced (24 March to 8 April, 2020). The implementation of the GeneXpert further reduced the median TAT to 4.8 h (8 April to 9 June, 2020). The GeneXpert system had the shortest median TAT (1.9 h), followed by the cobas (5.5 h) and by our custom platform (6.9 h).

**Conclusion:**

This work shows that the combination of high-throughput systems and rapid tests allows the efficient processing of a large number of tests with a short TAT. In addition, the use of a custom platform allowed the quick implementation of an in-house test when commercial assays were not yet available.

## Background

Coronavirus disease (COVID-19) is a disease caused by a novel coronavirus, the SARS-CoV-2, that initially appeared in the Wuhan area, China and was later declared a pandemic [1, 2]. In Switzerland, the first case was documented on 24 February 2020 and the disease spread and reached its peak on 23 March 2020 with 1454 new documented cases. Overall, 30 988 cases of COVID-19 were documented on 9 June 2020, with a total of 1633 deaths [3].

The microbiology laboratories were central in the response against COVID-19 as they had to quickly implement SARS-CoV-2 assays, to adapt to a sharp increase in the volume of tests and to maintain short turnaround times (TAT) [4–6]. Short TATs are indeed important to allow a quick tracing of cases, to optimize the use of scarce resources such as negative pressure rooms and to guide clinicians in patient management. However, the increasing amount of scientific literature on SARS-CoV-2 diagnostic assays has focused on assessment of their performance [7–12] and to our knowledge, this is the first publication to specifically address reducing the TAT of SARS-CoV-2 assays.

In this paper, we describe how the implementation of the different assays affected the TAT. This work also shows the impact of the sample type, analytical errors and of the result of the analyses on the TAT.

## Methods

We extracted information from all the SARS-CoV-2 analyses performed at CHUV (Lausanne University Hospital) from 24 February to 9 June, 2020. As our laboratory is a reference centre for COVID-19 testing, samples also originated from surrounding hospitals and screening centres. Information on the analyses included the sample type, the type of assays that were used (cobas, GeneXpert and/or our custom platform), their result and the different timestamps (time of reception at the pre-analytic laboratory and the time of the biomedical validation of the result). We excluded all analyses performed for quality control and all the analyses that were cancelled after their registration in our laboratory information system. The data were obtained during a quality enhancement project at our institution. According to the Swiss national law, conducting and publishing the results of such a project is permitted without ethics committee approval.

### Assays

The molecular diagnosis laboratory of the Lausanne University Hospital developed a custom platform for automated testing, as described elsewhere [13]. An in-house SARS-CoV-2 assay based on the work of Corman *et al*. [14] was implemented for this custom platform. This assay targets the E and the RdRp genes, but due to the low performances of the RdRp RT-PCR [7, 15], only the E gene RT-PCR was used after the first 893 samples.

The Roche SARS-CoV-2 assay for the cobas 6800 system [16] was implemented on 24 March 2020. The Cepheid GeneXpert Xpress SARS-CoV-2 assay [8] was implemented on 21 April 2020. Both assays showed perfect agreement with our in-house RT-qPCR, with kappa values of 0.98 (as published by Opota *et al*. [17]). Several publications showed similar results [8, 9, 11, 12]. The GeneXpert assay was however reported to have a better sensitivity for samples with a low viral load [8, 9].

### Organization

After reception at the pre-analytic laboratory, the samples first had to be registered in our laboratory information system, MOLIS (CompuGroup Medical, AG). The time of reception timestamp corresponds to the time of registration in MOLIS. Some analyses were prioritized over others: we prioritized samples from the emergency department, for patients in need of urgent surgery or samples tagged as urgent by the clinicians. Then came the samples from the different wards of the CHUV, from the external hospitals and finally, from the screening centres.

Initially, all analyses were performed on our custom platform. The analyses were progressively transferred onto the cobas system after its introduction. The GeneXpert system was used for specific cases (samples from the emergency department or in the context of pre-operative assessment for surgeries or organ transplantation).

Before being transmitted to the clinicians, all results had to be validated by a laboratory technician (technical validation) and a clinical microbiologist (biomedical validation). An automated validation by expert systems was progressively introduced to perform the biomedical validation. Its use was initially restricted to the validation of negative results, but starting from 8 April 2020 it was extended to validate all analysis. The results of our custom platform were automatically validated by FastFinder (UgenTec, Hasselt, Belgium). The results of the cobas and the GeneXpert systems were automatically validated by their accompanying expert systems [18].

### Statistics

All statistics were done with Python v3.7.3 [19] and the scipy package v1.1.0 [20]. The Mann-Whitney U test was used unless otherwise specified.

## Results

**Fig. 1.**
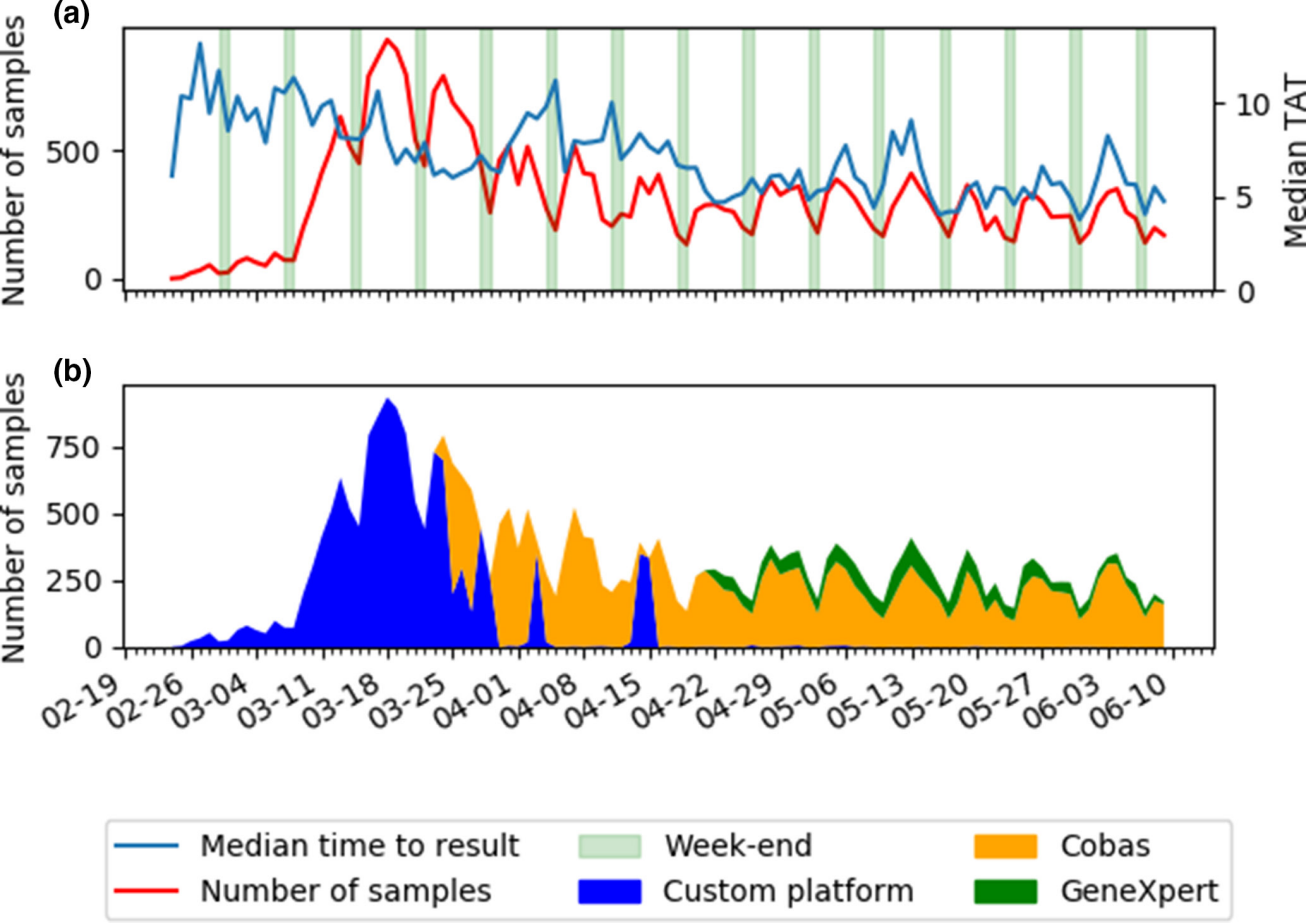
(a) Median turnaround time in hours and number of samples received per day (b) Samples in function of platform (adapted from [17, 23])

**Fig. 2.**
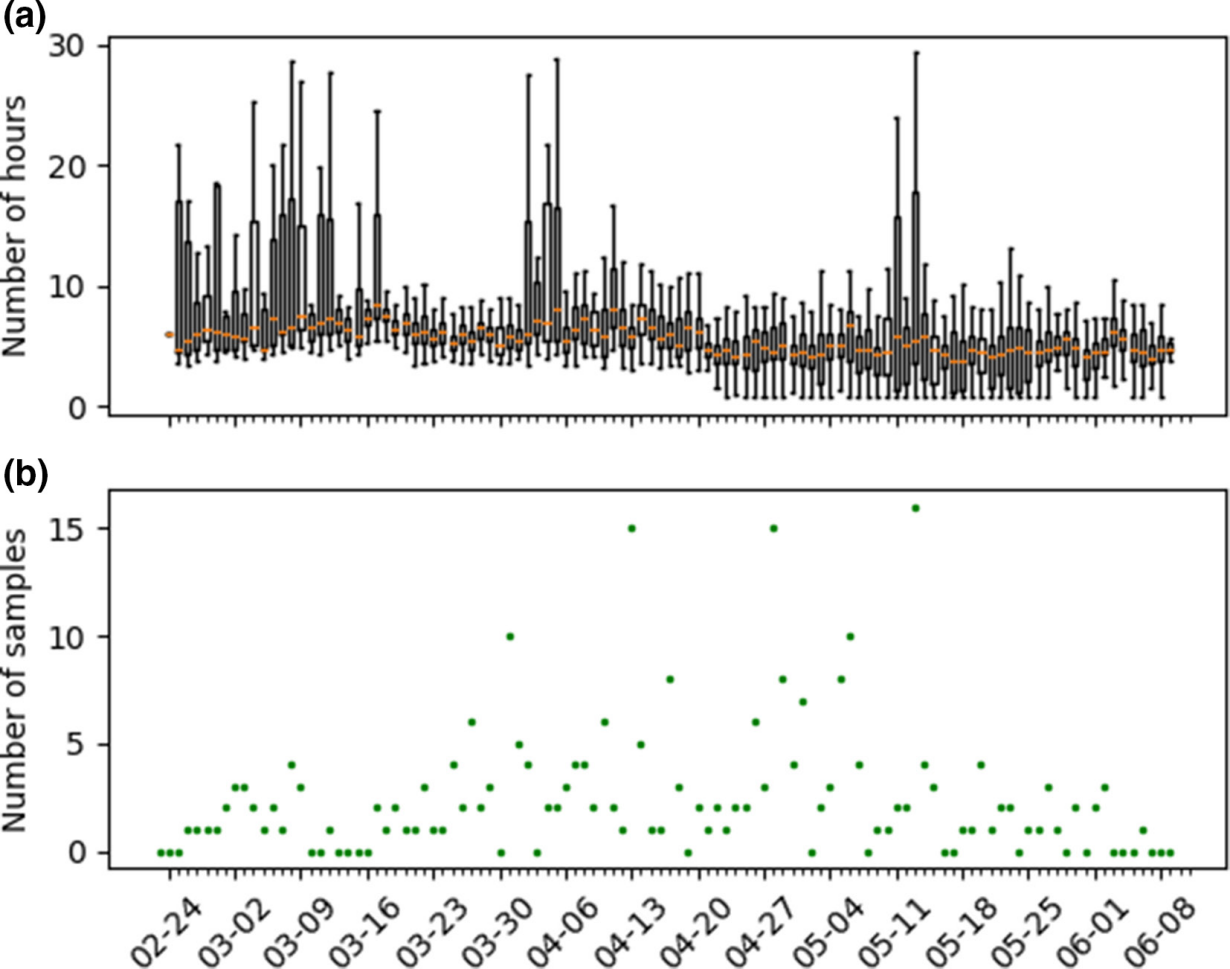
(a) Boxplot of the turnaround time (outliers not shown) (b) Number of samples with a turnaround time (TAT) >24 h.

We retrieved a total of 33 900 tests for analysis, with a median TAT of 6.25 h (range 0.9–678). Of those analyses, 18 153 (53.5 %) were performed on the cobas system (median TAT=5.5 h, range: 2.8–114.8), 12 941 (38.2 %) on our custom platform (median TAT=6.9 h, range 3.5–678), 2756 (8.1 %) on the GeneXpert system (median TAT=1.9 h, range: 0.8–53.0) and 50 (0.1 %) were performed on more than one platform (median TAT=7.4 h, range: 4.1–34.2). The cobas system allowed shorter TATs than our custom platform (5.5 vs 6.9, *P* <0.001). The GeneXpert system was faster than both the cobas (1.9 vs 5.5, *P* <0.001) and our custom platform (1.9 vs 6.9, *P* <0.001). The increase of the number of samples received per day is shown in Fig. 1(a). There was a mean number of 317 samples per day (range 2–933). Fig. 1(b) shows the repartition of the samples on the different diagnostic platforms. The median TAT when only our custom platform was in use was 6.9 h (range 3.5–297.5). It decreased to 6.1 h (*P* <0.001) after the introduction of the cobas system (range 2.9–678.1) and was further improved to 4.8 h (*P* <0.001) with the implementation of the GeneXpert system (range 0.8–408.4). The evolution of the TAT is shown in Fig. 2(a). Most analyses were performed on the cobas system after its introduction. Due to technical problems and maintenance (3 April and, 14–15 April 2020 respectively) of the cobas system, testing was temporarily transferred to our custom platform.

As shown in Tables 1 and S1 (available in the online version of this article), there were 267 samples (0.8 %) with a TAT longer than 24 h (median: 29.75; range 24.0–678.1), with a mean number of 2.5 such analyses per day (range 0–16). The number of analyses with a TAT longer than 24 h is shown in Fig. 2(b). The majority of those were nasopharyngeal swabs (117 of 267). Analysis with long TAT were however overrepresented in unusual samples like bone marrow (1 of 1), ophthalmologic samples (2 of 2), bile aspiration (1 of 1), blood (25 of 48) or CSF (33 of 45). The peaks of analyses with long TAT on 13 April, 28 April and 13 May, 2020 (Fig. 2b) were due to groups of external samples that had to be delayed due to our priority policy.

**Table 1.** TAT according to the platform, the results and the type of sample

Platform	Sample	No. of pos./Neg./Inv. (% pos)*	Median TAT for pos./Neg./Inv./All.	No. of samples with TAT >24 h (%)
**Overall**	**Total**	**4513/29216/171** (13.31 %)	**6.25/5.82/10.47/5.90**	**267** (0.79 %)
**cobas**	**Total**	**1740/16323/90** (9.59 %)	**5.65/5.43/10.44/5.47**	**91** (0.50 %)
	Nasopharyngeal swabs	1674/15811/73 (9.53 %)	5.62/5.43/10.62/5.45	69 (0.34 %)
	Respiratory samples†	54/330/9 (13.74 %)	7.13/6.09/9.90/6.37	25 (6.36 %)
	Other Samples‡	12/182/8 (5.94 %)	6.80/5.80/11.07/5.96	6 (2.97 %)
**Platform**	**Total**	**2709/10204/28** (20.93 %)	**6.65/6.90/27.88/6.85**	**172** (1.33 %)
	Nasopharyngeal swabs	2440/8730/11 (21.82 %)	6.58/6.82/19.68/6.77	54 (0.48 %)
	Respiratory samples†	260/1280/2 (16.86 %)	7.29/7.49/13.37/7.47	13 (0.84 %)
	Other samples‡	9/194/15 (4.13 %)	8.98/23.20/47.77/23.58	105 (48.17 %)
**GeneXpert**	**Total**	**64/2683/9** (**2.32 %**)	**1.51/1.28/2.70/1.28**	**3** (0.11 %)
	Nasopharyngeal swabs	63/2656/9 (2.31 %)	1.52/1.28/2.70/1.30	3 (0.11 %)
	Respiratory samples†	0/5/0 (0.00 %)	- / 1.72 / - / 1.72	0 (0.00 %)
	Other Samples‡	1/22/0 (4.35 %)	1.00/1.02/- / 1.02	0 (0.00 %)
**Multiple platforms**	**Total**	0/1/49 (0.00 %)	- **/ 18.68/7.33/7.37**	**1** (2.00 %)

*Result of the first analysis. Invalid analyses were repeated (the result of the repeated analysis are not shown).

†includes sputum, oropharyngeal, nasal and mouth swabs and bronchoalveolar lavages (BAL) and mini-BAL.

‡includes blood, urine, stools, anal swabs, bile, obstetrical samples, CSF and biopsies.

Some analyses had technical problems during the run and their result is referred to as invalid (due to the inhibition of the reaction [21, 22] or clotting [12]). There were 140 such results on the cobas system (0.8 %), 28 on our custom platform (0.2 %), and nine on the GeneXpert system (0.004 %). Repeating the analysis on leftover material allowed a conclusive result to be reached in most cases (cobas: 134 of 140; custom platform 28 of 28; GeneXpert: 9 of 9). Fifty of initially invalid cobas results were repeated on a different platform (GeneXpert in 38 of 50 and our custom platform in 12 of 50), allowing to reach a conclusive result in all cases.

Surprisingly, the result of the analyses also had an impact on the TAT: for the cobas and the GeneXpert systems, negative results had a shorter TAT than positive results (5.4 vs 5.7, *P* <0.001 and 1.3 vs 1.5, *P* <0.05 respectively). For the cobas system, this difference is due to the automatic results validation system (TAT of 5.5 h and negative=5.4 vs positive=6.0, *P* <0.001). The same comparison cannot be performed for the GeneXpert system, as all its results were automatically validated. The effect was opposite for our custom platform (negative: 6.9 vs positive: 6.7, *P* <0.001), however no cause could be determined for this effect.

**Fig. 3.**
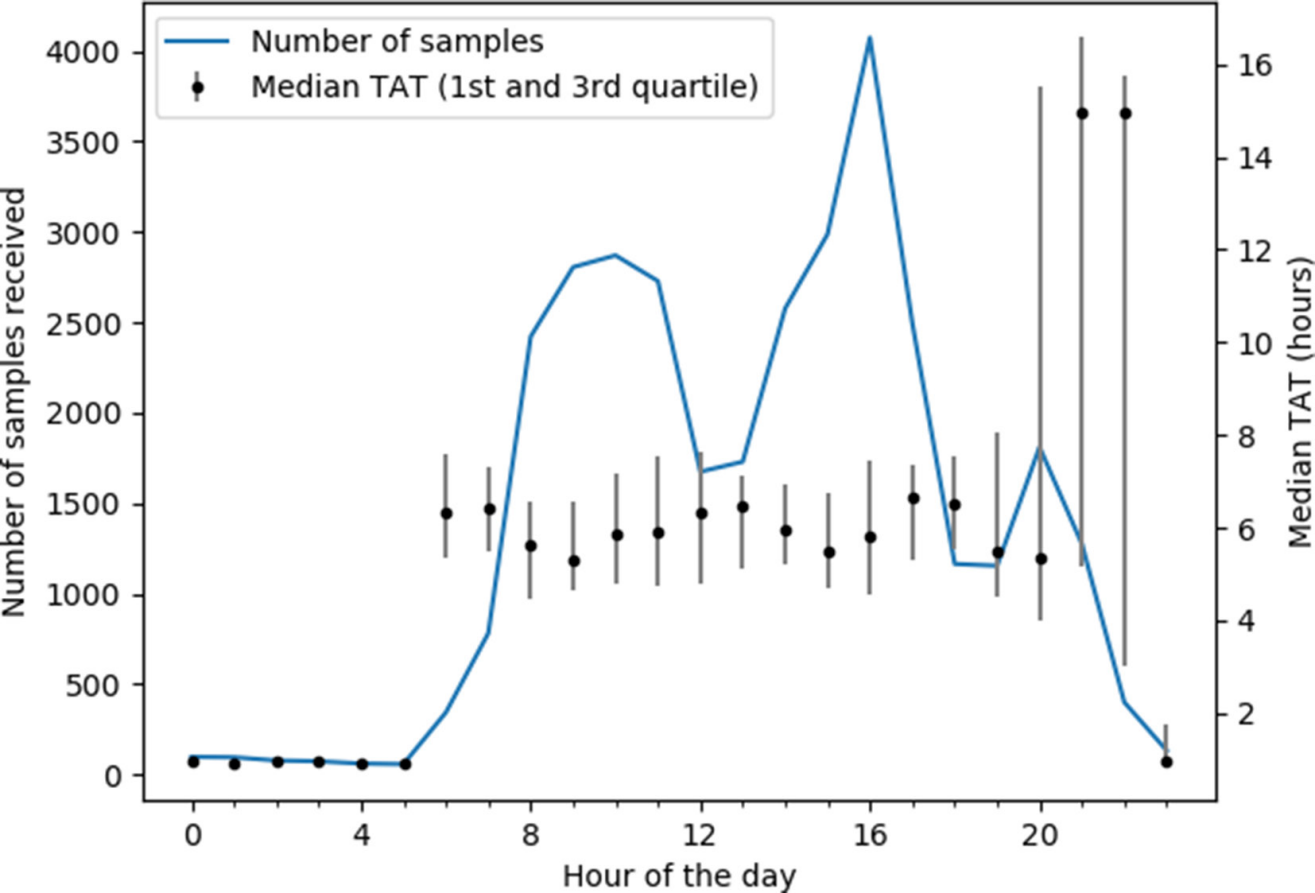
Number of samples received and turnaround time in function of the hour of the day

As shown in Fig. 3, most samples were received between 8 am and 9 pm, with three peaks centred at 10 am, 5 pm and 8 pm. The two last peaks correspond to samples from external hospitals that were received in batches. The median TAT was 5.9 h between 6 am and 8 pm, 15.0 h between 9 pm and 10 pm (late arrivals from the wards and the external hospitals that were performed the next day) and 1.0 h between 11 pm and 5 am (samples from the Emergency department performed on the GeneXpert).

## Discussion

This work aimed to study the impact of different analysis platforms on the TAT.

These results show that the association of a high-throughput system like the cobas system and a faster system for individual samples like the GeneXpert system allows short turnaround times. As already noted [13], the additional flexibility gained by using a custom platform allowed the quick implementation of a SARS-CoV-2 assay and to cope with a sudden increase in the number of tests, when the cobas SARS-CoV-2 assay was still not approved by the U.S. Food and Drug Administration. However, despite the automation of most steps of the qPCR, our custom platform was predictably slower than the cobas system, that fully automates all steps [16]. The complete automation on the cobas system also reduces the workload of laboratory technicians: as many as six technicians were necessary to operate our custom platform, while only two could achieve a similar throughput of 900 tests per day on the cobas system.

Additionally, in the context of a global shortage of reagents, pipet tips and PCR plates, it is important to maximize testing efficiency. However, this may come at the cost of longer TATs as samples which arrive early may be held until sufficient numbers are reached to maximize use of a PCR plate. Assuming the minimum TAT for the cobas and the custom platform (2.9 and 3.5 h, respectively) is close to the optimal TAT, this shows that a significant proportion of the TAT is spent waiting run completion (median of 5.5 h vs an optimal 2.9 h for the cobas and median of 6.9 h vs optimal 3.5 h for our custom platform). The use of smaller PCR plates may help mitigate the problem, if feasible. Additionally, having both the cobas and the custom platform was useful to alleviate shortage problems. Tips, reagents, processing plates or waste-covers for the cobas system were particularly impacted by such problems and made it necessary to rely on our custom platform despite slightly longer TATs. Having two high-throughput systems was helpful to cope with maintenance or unforeseen downtimes: we were able to transfer the samples from the cobas system to our custom platform without causing delays when the former had to be shutdown.

The introduction of the GeneXpert system allowed a faster track of analysis for urgent samples (such as an assessment for eligibility for an organ transplant), but shortages in reagents limited its widespread use. Additionally, operating the GeneXpert system does not require specialized technicians, which allows analyses for samples collected at nighttime to be run without delay.

While the choice of the analysis platform obviously affects the TAT, some other factors also have an impact: counter-intuitively, using an automated system to validate the analysis delayed the validation of positive results as compared to a manual validation, due to long running times. Inevitably, prioritizing some samples over others may lengthen the TAT of others, and in our case, it caused the three peaks in the number of TAT. The priority policy should be carefully planned. Overall, analysis with TAT longer than 24 h were rare and were over-represented in unusual samples.

## Conclusion

This work shows the result of using a combination of high-throughput systems for the bulk of the analysis and a faster system for selected individual samples. The former allows a high volume of analyses and the latter allows shorter TAT for urgent samples. With this organization, we achieved a median TAT of 6.25 h. Overall, TATs were shorter than 8 h in 82.1 % of the cases, less than 12 h in 89.3 % of cases and less than 24 h in 99.2 % of cases.

## Supplementary Data

Supplementary material 1
