# Supplementary material for: Impact of different SARS-CoV-2 assays on laboratory turnaround time
Source: J Med Microbiol. 2021 May 6;70(5):001280. doi: 10.1099/jmm.0.001280 (PMC8289200; doi:10.1099/jmm.0.001280)
Supplement: Supplementary material 1 [file jmm-70-1280-s001.pdf]

Supplementary material

Platform	Sample	Number of Pos./Neg./Inv. (% pos)	Median TAT for Pos./Neg./Inv./All.	Number of samples with TAT>24h
**Overall**	**Total**	**4513/29216/171 (13.31)**	**6.25 / 5.82 / 10.47 / 5.90**	**267 (0.79%)**
**Cobas**	**Total**	**1740 / 16323 / 90 (9.59)**	**5.65 / 5.43 / 10.44 / 5.47**	**91 (0.50%)**
	Nasopharyngeal swabs	1674 / 15811 / 73 (9.53)	5.62 / 5.43 / 10.62 / 5.45	60 (0.34%)
	Upper resp. samples[1]	23 / 101 / 6 (17.69)	7.23 / 6.83 / 9.48 / 7.13	9 (6.92%)
	BAL	5 / 53 / 0 (8.62)	8.87 / 7.58 / - / 7.59	7 (12.07%)
	Sputum	8 / 28 / 1 (21.62)	9.68 / 11.68 / 16.45 / 11.13	9 (24.32%)
	Nasal swabs	7 / 17 / 0 (29.17)	5.90 / 4.68 / - / 5.33	0 (0.00%)
	Oropharyngeal swabs	9 / 100 / 0 (8.26)	4.37 / 5.07 / - / 5.03	0 (0.00%)
	Anal swabs	12 / 178 / 8 (6.06)	6.80 / 5.80 / 11.07 / 5.96	5 (2.53%)
	Mini-BAL	0 / 9 / 2 (0.00)	- / 6.00 / 12.91 / 6.17	0 (0.00%)
	Pleural fluid	0 / 1 / 0 (0.00)	- / 44.43 / - / 44.43	1 (100.00%)
	Mouth swabs	2 / 22 / 0 (8.33)	6.03 / 4.82 / - / 4.82	0 (0.00%)
	CSF	0 / 1 / 0 (0.00)	- / 7.08 / - / 7.08	0 (0.00%)
	Obstetrical samples[2]	0 / 1 / 0 (0.00)	- / 4.23 / - / 4.23	0 (0.00%)
	Stools	0 / 1 / 0 (0.00)	- / 5.18 / - / 5.18	0 (0.00%)
**Platform**	**Total**	**2709 / 10204 / 28 (20.93)**	**6.65 / 6.90 / 27.88 / 6.85**	**172 (1.33%)**
	Nasopharyngeal swabs	2440 / 8730 / 11 (21.82)	6.58 / 6.82 / 19.68 / 6.77	54 (0.48%)
	Nasal swabs	247 / 1234 / 2 (16.66)	7.28 / 7.48 / 13.37 / 7.45	2 (0.13%)
	Oropharyngeal swabs	2 / 10 / 0 (16.67)	7.24 / 5.93 / - / 5.93	0 (0.00%)
	Anal swabs	4 / 32 / 4 (10.00)	6.08 / 7.75 / 15.91 / 8.15	5 (12.50%)
	Blood	1 / 45 / 2	10.40 / 24.82 / 57.73 / 25.10	25 (52.08%)

		(2.08)		
	Urine	0 / 7 / 0 (0.00)	- / 9.58 / - / 9.58	1 (14.29%)
	Mouth swabs	4 / 2 / 0 (66.67)	11.23 / 7.33 / - / 8.28	1 (16.67%)
	Sputum	2 / 14 / 0 (12.50)	9.82 / 13.93 / - / 12.53	5 (31.25%)
	BAL	2 / 11 / 0 (15.38)	28.88 / 11.80 / - / 11.80	4 (30.77%)
	Mini-BAL	1 / 1 / 0 (50.00)	13.88 / 102.23 / - / 58.06	1 (50.00%)
	Vaginal swabs	0 / 2 / 0 (0.00)	- / 9.02 / - / 9.02	0 (0.00%)
	Obstetrical samples[2]	3 / 33 / 0 (8.33)	8.98 / 20.57 / - / 18.64	16 (44.44%)
	CSF	0 / 43 / 1 (0.00)	- / 50.18 / 47.77 / 50.16	33 (75.00%)
	Skin swabs	0 / 6 / 3 (0.00)	- / 23.43 / 47.10 / 30.08	5 (55.56%)
	Tissue samples	0 / 7 / 2 (0.00)	- / 30.15 / 85.92 / 43.50	6 (66.67%)
	Upper resp. samples[1]	2 / 8 / 0 (20.00)	5.72 / 8.97 / - / 7.49	0 (0.00%)
	Pleural fluid	0 / 2 / 0 (0.00)	- / 58.73 / - / 58.73	2 (100.00%)
	Stools	1 / 16 / 0 (5.88)	76.00 / 23.23 / - / 23.53	8 (47.06%)
	Bone marrow	0 / 0 / 1 (0.00)	- / - / 408.37 / 408.37	1 (100.00%)
	Ophthalmologic samples[3]	0 / 0 / 2 (0.00)	- / - / 69.67 / 69.67	2 (100.00%)
	Bile	0 / 1 / 0 (0.00)	- / 28.20 / - / 28.20	1 (100.00%)
**GeneXpert**	**Total**	**64 / 2683 / 9 (2.32)**	**1.51 / 1.28 / 2.70 / 1.28**	**3 (0.11%)**
	Nasopharyngeal swabs	63 / 2656 / 9 (2.31)	1.52 / 1.28 / 2.70 / 1.30	3 (0.11%)
	Anal swabs	0 / 5 / 0 (0.00)	- / 1.72 / - / 1.72	0 (0.00%)
	Nasal swabs	1 / 20 / 0 (4.76)	1.00 / 1.00 / - / 1.00	0 (0.00%)
	Oropharyngeal swabs	0 / 1 / 0 (0.00)	- / 1.05 / - / 1.05	0 (0.00%)
	Sputum	0 / 1 / 0 (0.00)	- / 6.23 / - / 6.23	0 (0.00%)
**Multiple platforms**	**Total**	**0 / 1 / 49 (0.00)**	**- / 18.68 / 7.33 / 7.37**	**1 (2.00%)**

Table S1. Complete table of results

[1]Includes bronchial or tracheal aspirations

[2]Includes samples from the placenta, from amniotic fluid or from fetal swabs

[3]Conjonctival biopsy and anterior chamber fluid
